# Nutraceuticals in Paediatric Patients with Dyslipidaemia

**DOI:** 10.3390/nu14030569

**Published:** 2022-01-27

**Authors:** Giuseppe Banderali, Maria Elena Capra, Claudia Viggiano, Giacomo Biasucci, Cristina Pederiva

**Affiliations:** 1 Paediatrics Unit, Clinical Service for Dyslipidaemias, Study and Prevention of Atherosclerosis in Childhood, ASST-Santi Paolo e Carlo, 20142 Milan, Italy; giuseppe.banderali@asst-santipaolocarlo.it (G.B.); claudia.viggiano@unimi.it (C.V.); cristina.pederiva@asst-santipaolocarlo.it (C.P.); 2 Paediatrics and Neonatology Unit, Centre for Paediatric Dyslipidaemias, Guglielmo da Saliceto Hospital, 29121 Piacenza, Italy; m.capra@ausl.pc.it; 3 Department of Translational Medical and Surgical Sciences, University of Parma, 43126 Parma, Italy

**Keywords:** nutraceuticals, paediatric, diet, familial hypercholesterolaemia, dyslipidaemia

## Abstract

Coronary heart disease (CHD) is the main cause of death and morbidity in the world. Childhood is a critical period during which atherosclerosis may begin to develop; in the presence of familial hypercholesterolaemia (FH), the lifelong elevation of LDL cholesterol levels greatly accelerates atherosclerosis. Lowering LDL-C levels is associated with a well-documented reduction in cardiovascular disease risk. Current guidelines support the dietary and lifestyle approach as the primary strategy of intervention in children and adolescents with FH. Nutraceuticals (functional foods or dietary supplements of plant or microbial origin) are included in the EU guidelines as lifestyle interventions and may provide an additional contribution in reducing LDL levels when pharmacological therapy is not yet indicated. Meta-analyses of randomised clinical trials have demonstrated that the same nutraceuticals improve lipid profile, including lowering LDL-C, total cholesterol and triglyceride levels. In this narrative review, starting from current scientific evidence, we analyse the benefits and limitations of the nutraceuticals in children and adolescents with dyslipidaemia, and we try to evaluate their use and safety in clinical practice.

## 1. Introduction

Coronary heart disease (CHD) is the first cause of mortality and morbidity worldwide, especially in industrialised countries: in Europe, cardiovascular events account for 45% of mortality in adults, with a prevalence of 49% in females and 40% in males [1].

Dyslipidaemia is an important cardiovascular risk factor: high total cholesterol (TC) and low-density lipoprotein cholesterol (LDL-C) levels, low high-density lipoprotein cholesterol (HDL-C) and elevated triglycerides (TG) levels support the atherosclerotic pathways from the first decades of life. An altered lipid profile is a common finding both in the adult and paediatric population, with an estimated prevalence of 10–25% in children and adolescents. Dyslipidaemia is constantly increasing in children and adolescents, together with the epidemic increase of overweight and obesity [2].

It is recognised worldwide that the atherosclerotic process begins early in life and progresses through childhood into adulthood. The cumulative risk of exposure to elevated LDL-C levels accelerates the progression of atherosclerosis: the longer the exposure to high LDL-C, the higher is the CHD risk. In patients with homozygous familial hypercholesterolaemia (HoFH), LDL-C values are extremely elevated starting from birth: in these patients, myocardial stroke and angina pectoris have been described even before the age of ten years. Early detection and treatment of children and adolescents at high CHD risk is a fundamental milestone in CHD prevention: early intervention on dietary habits, lifestyle and, if necessary, pharmacological therapy started in paediatric age can modify the natural history of the disease, thus “gaining decades of life” with a drastic reduction in CHD events in adulthood [3,4].

According to the main Guidelines and Consensus Statements (American Academy of Pediatrics, AAP and European Atherosclerosis Society, EAS), the first step in the treatment of hypercholesterolaemia in children and adolescents are nutritional and dietary approach and the improvement of the lifestyle, as shown in Table 1.

If dietary and lifestyle interventions are not effective on LDL-C reduction and/or in the presence of severe hypercholesterolaemia, pharmacological treatment should be considered [5,7]. 

The so-called functional foods have recently been introduced in the treatment of dyslipidaemia in adult patients. Dietary supplements or fortified foods have been widely used as a complement of the diet in adult patients with dyslipidaemia, and in the latest EAS guidelines [6], they have also been indicated for paediatric patients with familial hypercholesterolaemia (FH).

The term “nutraceutical” was coined from “nutrition” and “pharmaceutical” in 1989 by Stephen DeFelice and can be defined as “a food (or part of the food) that provides medical or health benefits, including the prevention and/or treatment of a disease”.

**Figure 1:**
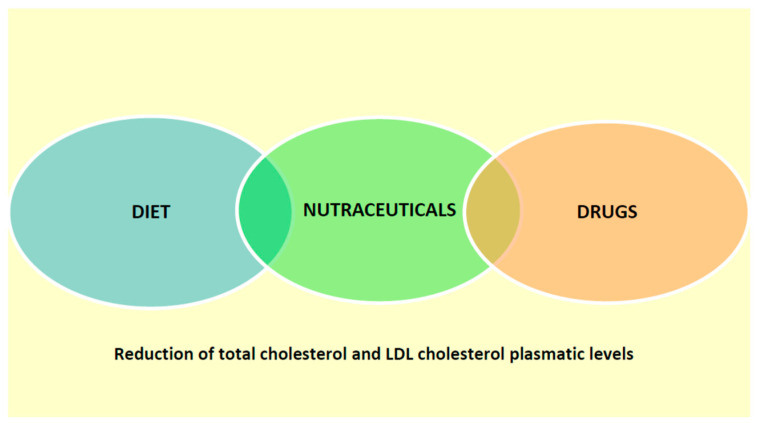
Use of nutraceuticals in dyslipidaemia in childhood.

We have schematically represented the possible use of nutraceuticals in the treatment of dyslipidemia in pediatric age in Figure 1.

Based on the current scientific evidence, this narrative review aims to analyse the benefits and limitations of nutraceuticals in children and adolescents with dyslipidaemia and tries to evaluate their use and safety in clinical practice.

We decided to consider the most widely used nutraceuticals in dyslipidaemia treatment. We started from each nutraceutical mechanism of action, and we considered the most important studies conducted on adult subjects. Moreover, we analysed the existing evidence in childhood and reported the main indications for paediatric patients in the present consensus documents.

## 2. Nutraceuticals and Dyslipidaemias

Nutraceuticals are included in the recent guidelines as lifestyle interventions for adult patients with dyslipidaemia in order to reduce total-cholesterol, LDL-cholesterol and triglycerides plasma levels, especially in those patients with moderately altered lipid profile, as primary prevention, when pharmacological intervention is not yet indicated [6]. Nutraceuticals showed a moderate lipid-lowering action, and their use in adult patients is safe and often well tolerated. They exert their lipid-lowering effect through different mechanisms: inhibition of cholesterol absorption, synthesis and metabolism, thus obtaining a multiple-line intervention, which can be combined with dietetic and lifestyle treatment, other nutraceuticals and pharmacological therapy [8]. Nutraceuticals have multiple pleiotropic effects: they enhance endothelial function, modulate arterial wall stiffness and have anti-inflammatory and anti-oxidative properties [9]. They are often well tolerated also by those patients that are intolerant to statin therapy [10]. Old patients (aged > 75 years) and patients that, despite therapy with statin or ezetimibe, cannot reach LDL-cholesterol target levels could benefit from the association of pharmacological therapy with nutraceuticals [11,12]. According to this evidence, in EAS guidelines [6], nutraceuticals are indicated as lipid-lowering agents for specific categories of both adult and paediatric dyslipidaemic patients above six years of age.

There are few randomised controlled studies, often based on small cohorts, analysing the effect of nutraceuticals in paediatric patients with dyslipidaemia. Fibres and phytosterols/stanols are the most widely studied nutraceuticals in childhood, whereas clinical trials concerning red yeast, soy proteins, probiotics, omega-3 fatty acids and nuts are sporadic and isolated [13].

Nutraceuticals with lipid-lowering effect can be divided into three main categories, according to their action on cholesterol metabolism:

- Inhibitors of intestinal cholesterol absorption;
- Inhibitors of liver cholesterol synthesis;
- Inducers of cholesterol excretion.

However, many nutraceuticals can act through multiple pathways and often with unclear mechanisms of action, with a final positive effect on lipid metabolism and atherosclerosis reduction, as shown in Table 2 [14].

## 3. Nutraceuticals Inhibitors of Intestinal Cholesterol Absorption

### 3.1. Fibres

Alimentary fibre is a component of plant foods constituted by carbohydrates resistant to the digestive process in the gastrointestinal tract. Alimentary fibre includes non-amylaceous polysaccharides (cellulose, hemicellulose, gum, pectin), oligosaccharides (inulin, fructo-oligosaccharides) and lignin.

From a functional point of view, alimentary fibre can be divided into four classes [15]:

(1) Insoluble fibre (bran): fibre not soluble in water and poorly fermented in the gut, with a possible mechanical laxative effect.
(2) Soluble fibre (inulin, dextrin, oligosaccharides), non-viscous, rapidly fermented; it does not cause any increase in viscosity and is completely fermented by gut microbiota; it can exert a prebiotic effect, without any laxative effect.
(3) Viscous soluble fibre, rapidly fermented (β-glucan, guar gum, pectin); it creates a viscous gel in water, thus increasing chime viscosity and consequently slowing nutrients absorption. It is quickly fermented in the gut, losing its laxative effect.
(4) Soluble viscous non-fermentable fibre (psyllium, multicellulose): it reduces nutrients absorption thanks to its viscosity and can exert a laxative effect.

**Figure 2:**
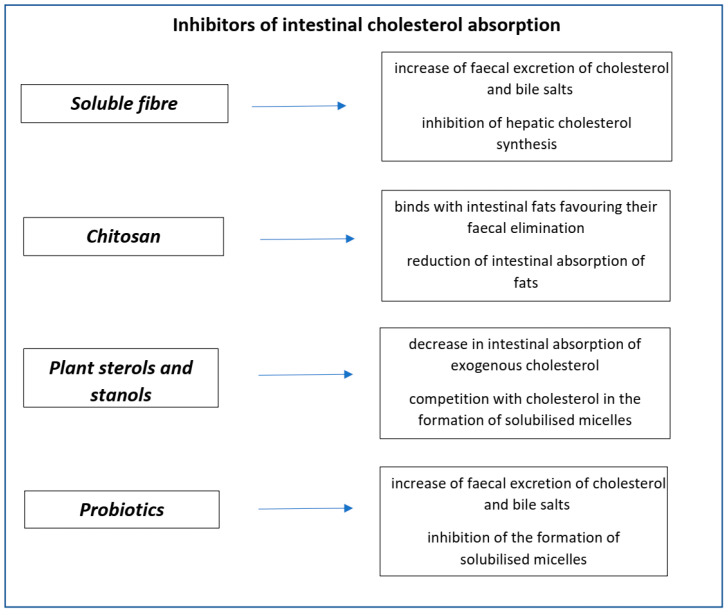
Nutraceuticals’ inhibitors of intestinal absorption.

The main characteristics of inhibitors of intestinal cholesterol absorption are summarised in Figure 2.

The cholesterol-lowering effect of fibre is mainly due to its viscosity: viscous fibres soluble in water form a gel that binds to bile salts in the gut and increases their excretion with stools. Cholesterol is one of the main components of bile; therefore, an increase in bile salts excretion causes an increased cholesterol use for hepatic bile synthesis. The higher the fibre viscosity, the greater its cholesterol-lowering effect [16]. Moreover, short-chain fatty acids (SCFA), which are derived from gut fermentation of fibre, can have positive effects on lipid profile [17].

The effect of fibre on lipid metabolism has been demonstrated in terms of total and LDL-cholesterol plasma levels reduction [18], and therefore, it has been recognised by the European Food Safety Authority (EFSA) [19]. Observational studies have shown that the habitual assumption of fibre is associated with a reduction in cardiovascular risk [20]. In particular, every 10 g increase in fibre daily assumption (especially if derived from fruits and whole cereals) is related to a reduction of 14% of acute coronary events and a reduction of 27% of death derived for coronary events [21].

Numerous studies have evaluated the effect of fibre assumption on lipid levels [22,23]. Dietary supplementation with oat β-glucan [24,25], psyllium [26,27], pectin [28], guar gum, glucomannan [29] and hydroxypropyl methylcellulose [30] significantly reduces LDL-cholesterol levels.

Psyllium is derived from the peel of a seed called *Plantago Ovata*; if added to cereals, it does not alter their taste or consistency, thus granting good compliance [31].

Oat is a soluble and viscous alimentary fibre derived from *Avena Sativa*, rich in β-glucan [32].

Glucomannan is derived from a tuber called *Amorphophallus Konjac*; it is the fibre with the highest molecular weight and the greatest viscosity, able to reduce LDL-cholesterol levels more effectively than psyllium, bran [16], oat and barley [33].

Fibre assumption in childhood is a debated topic. Food and Drug Administration (FDA) advise an adequate fibre daily intake in relation to the total daily caloric intake (12 g/1000 Kcal), whereas the AAP relates it to age and weight [34]. In clinical practice, for children above three years of age, the recommended daily fibre intake (in grams) is equal to the sum of age in years plus 5 [35,36,37]. A large general paediatric study on a cohort of 5873 Japanese school children has evaluated that daily fibre intake is inversely related to total cholesterol plasma levels and to the development of overweight and obesity, confirming data already reported in adult patients [38,39].

Psyllium effect on lipid profile has been demonstrated in various studies [36,38,39]. In a 12-week randomised controlled trial in a cohort of 50 children with mild hypercholesterolaemia in CHILD I dietary treatment (lipid daily intake <30% total daily calories, saturated fatty acids daily intake ≤10% of total daily calories, cholesterol daily intake <300 mg) or in CHILD II dietary treatment (fatty acids daily intake 7% of total daily calories, cholesterol daily intake <200 mg), treatment with psyllium 3.2 g/day resulted in an 8.9% reduction of LDL cholesterol if compared to the control group receiving only dietary treatment [38]. In another study, in a cohort of 36 children with familial combined hypercholesterolaemia (FCH) on CHILD I dietary treatment, a daily intake of psyllium (2.5–10/day, depending on age) was associated with an 18% and 23% reduction of total cholesterol and LDL-cholesterol levels, respectively [37].

Glucomannan has been successfully tested in a 24-week trial in a cohort of 36 children (aged 6–15 years) with FH. All children were advised to follow CHILD I dietary treatment for one month, and then, they were given glucomannan (1–1.5 g/day, according to the patient’s weight). After one month of treatment, their lipid profile was improved, with a reduction in the levels of total cholesterol (5.1%), LDL cholesterol (7.3%) and non-HDL cholesterol (7.2%), compared to pre-treatment values [40]. The same results were found in another study: 40 children treated with glucomannan (2–3 g/day) had a reduction in LDL cholesterol of 30% in females and 9% in males [41]. The efficacy of glucomannan on lipid profile was also analysed in two meta-analyses. In the first one, in a cohort of 531 obese children, the authors did not report any reduction in LDL-cholesterol levels but a statistically significant reduction in triglycerides levels [29]. In the second study, LDL cholesterol was significantly reduced both in children and in adults [42].

Oat bran effect on lipid profile has been poorly investigated up to now, and there are no recommendations on its assumption for paediatric patients [43]. A cross-over randomised clinical trial analysed the effect of carob seed flour on lipid profile: in a 16-week period, 11 children with FCH, 10 controls and 17 adults were given 8–30 g/day of carob seeds flour, with a consequent 11–19% reduction of LDL-cholesterol levels [44].

Most of the studies carried out on the use of the fibre in paediatric patients highlighted good compliance to the proposed therapy, thanks to the good palatability of the nutraceutical; side effects as diarrhoea and abdominal pain were only occasionally reported [39,40]. However, safety data on long term fibre treatment in paediatric patients are still limited.

In conclusion, fibre intake should mostly derive from an adequate fruits and vegetable intake. In case of persistently elevated total and LDL-cholesterol levels despite good compliance to dietary treatment, supplementation with soluble fibre should be considered in order to improve the lipid profile, with no relevant adverse effects.

### 3.2. Phytosterols and Stanols

Phytosterols and stanols are plant-derived bioactive components structurally similar to cholesterol. Phytosterols are steroidal alkaloids that differ from cholesterol for their lateral chain, whereas stanols are 5α-saturated derived from phytosterols. Both these compounds are not synthesised by humans, so they have to be introduced with foods, such as fresh fruits, nuts, vegetables, seeds, cereals, pulses and vegetable oils [45].

The use of foods enriched with phytosterols was included in 2011 National Cholesterol Education Program (NCEP) guidelines for LDL-cholesterol reduction. The cholesterol-lowering effect of phytosterols is based on the reduction of the intestinal absorption of exogenous cholesterol: due to their structural homology to cholesterol, phytosterols compete with cholesterol in the formation of solubilised micelles and in the binding with Niemann-Pick C1 Like Protein (NPC1L1) in the enterocytes; this condition is favoured by the highly hydrophobic properties of phytosterols compared to cholesterol. Phytosterols present in the enterocytes are quickly excreted in the lumen by transporters ABCG5 and ABCG8, so plasma phytosterols concentrations are always very low, and they are promptly available in the gut to compete with cholesterol. Moreover, the presence of phytosterols in the intestinal lumen inactivates acyl-CoA-cholesterol-acyltransferase, limiting cholesterol entry into the lymphatic vessels and its transport to the liver. The reduction of intestinal cholesterol entrance and of chylomicrons transport to the liver reduces plasma LDL-cholesterol levels [46,47].

Transversal studies have demonstrated an inverse correlation between natural phytosterols intake and LDL-cholesterol plasma levels [48,49]. Randomised controlled intervention studies have validated the cholesterol-lowering effect of phytosterols: the intake of functional foods containing phytosterols significantly reduces total and LDL-cholesterol plasma levels with an average reduction of 8–10% both in hypercholesterolaemic and in healthy subjects [46]. The effect of phytosterols is dose-dependent for doses <3 g/day, whereas for doses higher than 3 g/day, there is a plateau effect without any further beneficial effect on lipid metabolism [14].

There is little evidence of the effect of phytosterols in paediatric patients; however, the available literature demonstrates that phytosterol intake is associated with a reduction in total cholesterol levels in children with mild hypercholesterolaemia [50] and in children with FH [51,52]. The supplementation with 1.2–2 g/day phytosterols in children with FH in CHILD I or CHILD II dietary treatment has determined a further 10% reduction of LDL-cholesterol levels. An increased dose of phytosterols (2.3 g/day) was associated with further LDL-cholesterol reduction [51,52]. Treatment with phytosterols and stanols is usually well tolerated, and no major adverse effects have been reported so far, even if long term follow-up data are not yet available [14].

### 3.3. Chitosan

Chitosan is derived from chitin deacetylation. Chitin is a polymer that protects insects and shellfish, granting hardness and resistance to their shells. Chitosan can bind to lipids, thus reducing their intestinal absorption and promoting their elimination with the stools [53].

A meta-analysis of six randomised controlled trials on 416 adult patients with hypercholesterolaemia concluded that chitosan has a significant effect on total cholesterol reduction (−11.6 mg/dL, *p* = 0.002), but no effect on the other lipid parameters [54]. On the contrary, other studies highlighted the effect of chitosan on LDL cholesterol, HDL cholesterol and triglycerides [55]. These contrasting data suggest the need for further studies to better understand the lipid-lowering effect of chitosan [14]. Chitosan is usually well tolerated; transient minor adverse effects, such as abdominal pain, vomiting or diarrhoea, have been seldom reported [56]. Based on the mechanism of action, chitosan use seems to be promising, but data regarding its effects in the paediatric age are still lacking.

### 3.4. Probiotics

Probiotics are vital micro-organisms that, when taken in adequate amounts, confer a health benefit to the host. In the last years, some trials have supported the clinical use of probiotics as lipid-lowering agents. However, available studies are heterogeneous in terms of length, strains of probiotics, dose, clinical characteristics of study participants and type of carriers [14]. The mechanisms of action of probiotics on lipid metabolism are still unclear and not yet fully defined. Probiotics seem to interfere with gut cholesterol, binding to it or incorporating it in their cell membrane [57]. Lactobacillus acidophilus and Lactobacillus bulgaricus contain some enzymes able to catalyse cholesterol transformation, promoting cholesterol excretion with stools [58]. Other probiotics reduce the entero-hepatic circulation of bile salts through the activation of bile salt hydrolase. Some strains of Lactobacilli and Bifidobacteria can enzymatically de-conjugate bile acids, increasing their excretion and promoting cholesterol systemic hepatic mobilisation for bile salts de novo synthesis [59]. Other probiotics can influence intestinal pH, micelle formation, cholesterol transport and the transport of lipoproteins and of cholesterol esters [60]. These mechanisms are only hypothetical, so additional data are necessary to confirm which probiotic has a better lipid-lowering effect.

In adult patients with hypercholesterolaemia, the administration of different probiotic strains caused a reduction in total and LDL-cholesterol levels [61].

In a randomised, double-blind, placebo-controlled, cross-over trial in a cohort of paediatric patients with hypercholesterolaemia (total cholesterol ≥ 90° centile for age and sex), the oral intake of a three-strain Bifidobacteria probiotic blend resulted in an improvement in the lipid profile with a reduction of 3.4% of total and of 3.8% of LDL cholesterol [62]. The use of probiotics is considered safe and with no adverse effects [14].

## 4. Nutraceuticals Inhibitors of Liver Cholesterol Synthesis

**Figure 3:**
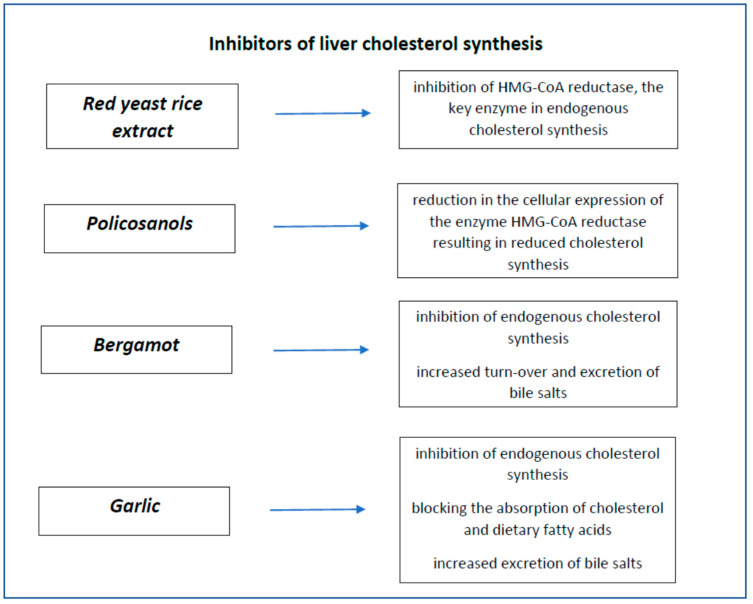
Nutraceuticals’ inhibitors of liver cholesterol synthesis.

Nutraceuticals that mainly act through the inhibition of hepatic cholesterol synthesis are red yeast rice, policosanols, bergamot and garlic. Their characteristics are summarised in Figure 3.

### 4.1. Red Yeast Rice

Red yeast rice (RYR) is obtained by the fermentation of a specific yeast (*Monascus purpureus*, *M. pilosus*, *M. floridanus*, *M. ruber*) in rice (*Oryza sativa*); it has been widely used in China in the past centuries to flavour foods [63].

Monascus purpureus ferments red rice and produces a substance called Monacoline K, which can inhibit the hepatic activity of HMGCoA reductase and, therefore, endogenous cholesterol synthesis. Monacolin K is structurally and functionally similar to lovastatin [64]. Cholesterol-lowering effect of RYR is only partially due to Monacolin K, as RYR contains at least other ten different kinds of monacolin, phytosterols able to reduce cholesterol gut absorption, fibres and niacin, that can have a lipid-lowering effect as well [63,64].

RYR has been proven to be effective and safe in patients with mild or moderate hypercholesterolaemia. The first prospective, double-blind, placebo-controlled study on the effect of RYR on lipid profile was conducted in the USA in 1999. Patients with hypercholesterolaemia not on pharmacological treatment were randomised to receive RYR 2.4 g/day or placebo for 12 weeks; at the end of the study, LDL-cholesterol levels were reduced by 22% compared to a reduction of 5% in the placebo group, with no significant side effects [65]. The efficacy and safety of RYR have been recently confirmed in a meta-analysis involving 20 studies: LDL-cholesterol reduction in a 2–24 months period of treatment was comparable to that obtained with a moderate-intensity statin (pravastatin 40 mg, simvastatin 10 mg or lovastatin 20 mg), and the incidence of hepatic, muscular or kidney adverse effects was similar to those reported in the placebo group [66].

RYR is one of the few nutraceuticals also studied to determine its efficacy in the secondary prevention of cardiovascular events: patients treated with RYR had a 20% reduction in LDL-cholesterol plasma levels and a 45% reduction of coronary events compared with the placebo group [67].

Commercial products containing RYR are still under analysis for their safety profile, as there is a large variety in the content of monacolin K, and the presence of a mycotoxin called citrinin has been reported [68,69].

According to EFSA claim in 2011, RYR with content of monacolin K ≤10 mg/die can be used in adult patients at mild to moderate cardiovascular risk, with LDL cholesterol higher no more than 25% of the therapeutic goals, despite dietary and lifestyle interventions [6,70].

There are few studies on the use of the RYR in paediatric patients. In a clinical trial conducted by Guardamagna et al., a paediatric population aged 8–16 years with mild to moderate dyslipidaemia treated for 8 weeks with RYR containing 3 mg/day of monacolin K showed an 18.5% and 25.1% reduction of the total and LDL cholesterol, respectively; no particular side effects were reported. However, monacolin is similar to lovastatin; therefore, a careful clinical and biochemical follow-up is mandatory when it is used in paediatric patients. The purity of the product and the absence of citrinin must be granted as well [69].

In conclusion, RYR could be a therapeutic alternative for paediatric patients with hypercholesterolaemia at high risk, but it must be accompanied by close clinical and biochemical monitoring under medical supervision [70,71].

### 4.2. Policosanols

Policosanols (PCS) are a mixture of long-chain alcohols extracted from plant waxes, sugar cane, rice bran and potatoes [72]. PCS have been widely used in Cuba in the last decades as lipid-lowering agents. Several studies demonstrated that PCS derived from sugar cane have better lipid-lowering activity than phytosterols, similar to statins, but with a better effect on HDL-cholesterol level and few side effects [73]. The mechanism of action of PCS is yet partially unknown, and it is probably due to a reduction in cellular expression of HMGCoA reductase, with a consequent reduction in cholesterol synthesis. The cholesterol-lowering effect seems to be dose-dependent in a dose range from 2 to 40 mg/day [74]. Recently, the lipid-lowering effect of PCS has been questioned in studies conducted in Europe and in the USA [75]. In 2011, EFSA rejected a claim in favour of PCS for lack of evidence [76].

There are few data on the use of PCS in paediatric patients with dyslipidaemia. In a study conducted by Guardamagna et al., the association of PCS and RYR showed a reduction of the total- and LDL-cholesterol levels, with good compliance and few adverse effects [71]. In conclusion, there is no evidence on efficacy and safety of PCS in paediatric patients, and PCS are not currently recommended in children [76].

### 4.3. Bergamot

Bergamot is the common name of Citrus bergamia Risso, a citrus rich in flavonoids, some of which have a statin-like lipid-lowering activity as they inhibit HMGCoA reductase in the liver [77]. Bergamot also has an anti-oxidative effect, and it can promote cholesterol stool excretion by reducing intestinal cholesterol absorption and increasing bile acid turnover [78]. There are few studies on the lipid-lowering effect of bergamot. Gliozzi et al. reported a reduction in LDL-cholesterol plasma levels (comparable to that obtained with rosuvastatin 10 mg/day) in patients with dyslipidaemia treated with 1000 mg/day bergamot for a four-week period [79]. In another study, a dose-dependent lipid-lowering effect was reported in patients treated with bergamot, both in patients with mild hypercholesterolaemia (LDL cholesterol: 130 mg/dL) and in those with combined dyslipidaemia (hypercholesterolaemia and hypertriglyceridaemia), with no adverse effects [80].

Up to now, studies with bergamot supplementation with doses ranging from 500 mg to 1500 mg/day have a good safety profile and no side effects reported [78]; this makes it interesting for possible use in children, but there is a lack of specific data in the literature.

### 4.4. Garlic

Garlic (*Allium sativum*) is a functional food with multiple health benefits. Garlic’s main active compound is called allicin. When fresh garlic is chopped or crushed, the enzyme alliinase converts alliin into allicin, which is responsible for the aroma of fresh garlic; the allicin generated is unstable and quickly changes into a series of other sulfur-containing compounds such as diallyl disulfide. Allicin can inhibit HMGCoA reductase, reducing endogenous cholesterol synthesis [81].

A meta-analysis of 39 RCT has reported garlic intake in a two-month period in patients with mild to moderate hypercholesterolaemia and showed a 9 mg/dL reduction of LDL-cholesterol levels [82]. Another study highlighted a good antihypertensive effect of garlic [83], whereas Jung et al. reported a significant association between garlic intake and improvement in LDL/apoB ratio with the reduction in apoB [84]. Garlic also has anti-platelets properties [85].

In conclusion, a 6 g garlic daily intake can be useful in the treatment of moderate hypercholesterolaemia, probably mainly due to garlic anti-platelets and anti-hypertensive properties rather than a direct lipid-lowering effect. Garlic is usually well tolerated, and adverse effects are mild, mainly gastrointestinal. However, the high dosage required and peculiar garlic aroma often interfere with patients’ compliance [86]. There is no available evidence in paediatric patients.

## 5. Nutraceuticals Inducer of LDL Cholesterol Excretion

Some nutraceuticals exert their lipid-lowering action through an increase in LDL-cholesterol excretion, promoting an increase in LDL-receptor expression and its half-life on the hepatocyte surface.

Soy, lupins and berberine are the most studied among this class of functional foods. Green tea extracts are in this category as well.

**Figure 4:**
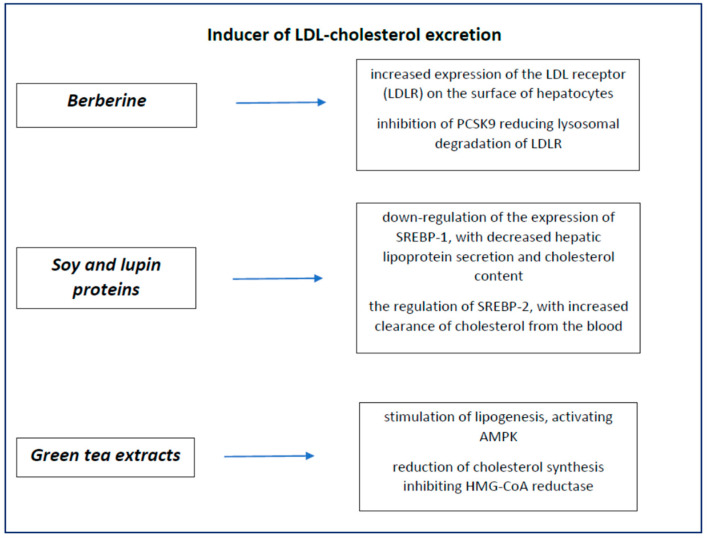
Nutraceuticals’ inducer of LDL-cholesterol excretion.

Their main characteristics are summarised in Figure 4.

### 5.1. Berberine

Berberine is a quaternary ammonium salt from the protoberberine group of benzylisoquinoline alkaloids found in such plants as Berberis, such as *Berberis vulgaris* (barberry), *Berberis aristata* (tree turmeric), *Mahonia aquifolium* (Oregon grape), *Hydrastis canadensis* (goldenseal), and *Coptis chinensis* (Chinese goldthread) [87]. Berberine is usually found in the roots, rhizomes, stems and bark [87].

Berberine cholesterol-lowering effect is determined by several mechanisms. Berberine promotes an increase in the expression and in the half-life of LDL receptor (LDL-R) on hepatocyte surface [88]; it also enhances the increase of LDL-R promoter transcriptional activity and the stabilisation of its mRNA [89]. Moreover, in vitro studies show that berberine inhibits proprotein convertase subtilisin/kexin type 9 (PCSK-9) activity, reducing lysosomal LDL-R degradation and increasing its availably in the liver [90].

In the first study on the effect of berberine intake on lipid profile in a cohort of patients with hypercholesterolaemia, berberine intake was related to a reduction both in LDL cholesterol and in triglycerides [89]. Berberine’s lipid-lowering effect has been analysed in three meta-analyses [91,92,93] that showed similar results: berberine allowed a 25 mg/dL reduction in LDL cholesterol, a significant reduction in triglycerides and a slight increase in HDL-cholesterol levels. Berberine main side effects, which occur at high dosages, are gastrointestinal, such as constipation, diarrhoea, abdominal pain and sour taste [93]; these characteristics make it unattractive in children.

### 5.2. Soy and Lupins

Soy is a bean (*Glycine max*) derived from an Asian plant, grown for its multiple nutritional properties; it is very rich in protein (36–46%), essential amino acids, lipids (18%), soluble carbohydrates (15%) and fibres (15%). Soy contains a lot of micronutrients, such as soy lecithin (0.5%), sterols (0.5%) and tocopherols (0.02%). Soy nutritional values and positive health effects have been studied for years, based on epidemiologic data suggesting an inverse relationship between soy intake and cardiovascular disease [94].

Soy lipid-lowering effect is mainly due to isoflavones, which increase LDL-R expression in the liver. Isoflavones can also bind to estrogen receptors, promoting an estrogen-like activity, thus affecting lipid metabolism directly through the modulation of the lipogenesis or indirectly influencing appetite and the energy balance [95]. Multiple mechanisms are involved in the lipid-lowering effect of the soy [96]: lecithin and sterols reduce the intestinal cholesterol absorption, β-glucan increases the bile salts excretion [96,97] with a consequent reduction in lipoprotein hepatic secretion, reduction in cholesterol synthesis, and increase in biliary acids stool excretion [98,99,100]. Bioactive peptides, such as gamma conglutinine (Cy), present both in soy and in lupins, have a lipid-lowering effect through LDL-R activation in the liver [101].

Descovich et al. described a reduction in total cholesterol levels in a cohort of 127 patients with hypercholesterolaemia treated with soy proteins [96]. Various meta-analyses have confirmed the soy lipid-lowering effect. In 2015, in a study involving 35 clinical trials with a total of 2670 patients, soy intake was associated with a reduction of 2% in total cholesterol, 3% in LDL cholesterol and 4% in triglycerides and an increase of 3% in HDL-cholesterol levels [102].

Lupins are beans with low salt content, low glycaemic index and no phytoestrogens. Lupins are composed of proteins (30–35%), fibres (30%), carbohydrates (3–10%) and lipids (6%), 81% of which are polyunsaturated fatty acids.

Lupins’ lipid-lowering effect has been described in several studies. Baehr et al. reported a positive effect on lipid profile (increase in HDL cholesterol and reduction in LDL cholesterol) in 33 subjects with hypercholesterolaemia treated with lupins (25 g/day) for a two-month period, followed by one month of wash-out [103].

There are few studies on soy and lupin effect on the lipid profile in paediatric patients. In a group of 16 children with FH on the CHILD I diet, the intake of soy milk (0.25–0.5 g/kg/day) was associated with a reduction in total cholesterol, LDL cholesterol and Apolipoprotein B (7.7%, 6.4% and 12.6%, respectively) [104].

In a recent study, the effect of soy intake on lipid profile was analysed in a cohort of paediatric patients with FH treated for a 13-week period. In the intervention group, LDL-cholesterol levels were reduced by 10% compared to pre-treatment values [105].

Chronic use of high quantity of soy, rich in isoflavones, could interfere with thyroid function and with fertility. Moreover, soy and its derivates are rich in phytic acid, which can reduce calcium, magnesium, copper, iron and zinc absorption.

Lupins have a good safety profile, and minor adverse effects have been reported. However, lupins’ lipid-lowering action is dose dependent, and this could interfere with long-term compliance [14].

### 5.3. Green Tea Extracts

Some trials on green tea suggest that it can have a protective effect on cardiovascular disease [106]. Green tea is rich in antioxidants, such as polyphenols, which are cardioprotective compounds. Furthermore, green tea can promote lipogenesis through AMP-activated protein kinase (AMPK) activation, and it can reduce cholesterol synthesis through HMGCoA reductase inhibition. Green tea may also interfere with micelle formation, thus reducing intestinal cholesterol absorption [14]. Green tea catechins have an inhibitory effect on apical biliary salt transporter in the ileum, so they can reduce bile salts re-uptake and increase liver expression of LDL receptors [107].

Some studies have reported the efficacy of green tea extracts on LDL cholesterol and on systemic blood pressure reduction [108]. Green tea is usually well tolerated, but if it is used for a long time and at a high dose, it can interfere with the intestinal absorption of iron and folate; for this reason, its use in children is unsafe.

## 6. Nutraceuticals with Mixed Action

**Figure 5:**
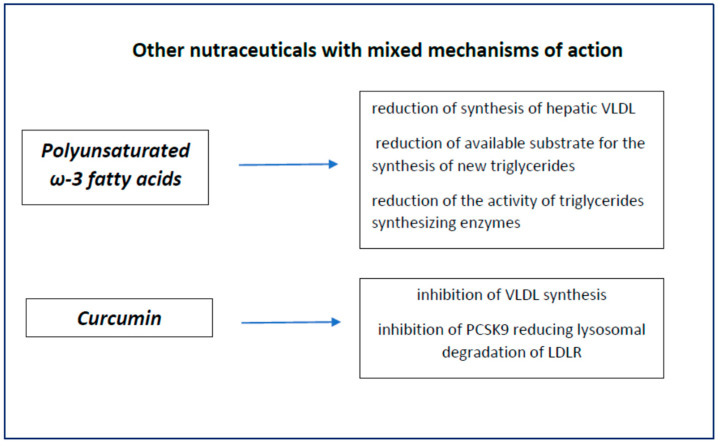
Nutraceuticals with mixed action.

In this category, we have nutraceuticals that exert their lipid-lowering and anti-atherogenic action with multiple mechanisms, often not completely known. Polyunsaturated long-chain fatty acids and curcumin are two of the most studied ones. Their characteristics are shown in Figure 5.

### 6.1. Omega-3 Polyunsaturated Long Chain Fatty Acids

Polyunsaturated fatty acids (PUFA) are fatty acids that have more than one double C=C bond in their molecule. With regard to cardiovascular prevention, omega-3 and omega-6 PUFA are the most studied, as they can modulate triglycerides, LDL cholesterol and inflammatory markers implied in the atherosclerotic process [109,110,111].

Omega-3 fatty acids are present in nature both in animals, such as fish, krill, eggs and squid, and in vegetables, such as seaweeds, nuts, flax seeds and sage. Omega-3 PUFA have positive effects on cardiovascular health, as suggested by epidemiological and intervention studies. In the last few years, EFSA [112], AHA (American Heart Association) [113] and FSANZ (Food Standard of Australia and New Zealand) [114] have recognised omega-3 PUFA as functional foods for cardiovascular disease. EFSA suggests that a 2 g/day supplementation with docosahexaenoic acid (DHA) and eicosapentaenoic acid (EPA) can maintain triglycerides plasmatic levels within normal range, whereas AHA suggests a 2–4 g/day supplementation with DHA and EPA to reduce triglycerides by 25–30% [113].

Omega-3 PUFA play their triglycerides lowering effect through different mechanisms: they reduce hepatic VLDL synthesis, act as false substrate in triglycerides synthesis, reduce the activity of enzymes involved in triglycerides synthesis, enhance fatty acids beta-oxidation and reduce fatty acids endogenous synthesis [115].

The positive effects of omega-3 fatty acids on cardiovascular risk factors have been confirmed by many authors. Eslick et al. published a very comprehensive meta-analysis involving 47 RCTs with approximately 16,500 patients with hypercholesterolaemia. The authors evaluated the effect on cardiovascular risk of a 3.5 g/day supplementation with EPA/DHA over 24 weeks; they found significantly reduced (14% compared to pre-treatment values) triglycerides, slightly reduced (2.5 mg/dL) LDL-cholesterol and unchanged HDL-cholesterol levels [116]. Later on, Leslie et al. confirmed these results in subjects with a normal lipid profile or with moderate dyslipidaemia, reporting a 9–26% reduction of triglycerides levels with a 4 g/day omega-3 PUFA intake and a 4–51% reduction with a 1–5 g daily intake [117].

The DART study, published in 1989, involved 2033 male patients with recent myocardial stroke randomised to different diets. After a two-year follow-up, the group treated with omega-3 showed a 29% reduction in mortality rate compared to the control group, and this reduction was mainly attributed to a decrease in cardiovascular events [118]. Approximately ten years later, in the GISSI study, 11,324 patients with recent myocardial stroke were enrolled. They were randomised to omega-3 PUFA and/or vitamin E for 3.5 years: after six months, no modification in the lipid profile was detected, while after twelve months, patients treated with omega-3 PUFA showed a 15% reduction both in global mortality and cardiovascular mortality (ictus cerebri and myocardial stroke); in the intervention group, death related to acute cardiovascular events was reduced by 45% [119]. In the JELIS study, the effect of EPA in addition to statin therapy was evaluated in 18,645 patients with hypercholesterolaemia: after 4.6 years follow up, a 19% reduction in major cardiovascular events was reported in the group that received EPA, but no modification on LDL or HDL cholesterol was detected [120]. These are the main trials analysing the hypothesis that supplementation with PUFA, alone or together with pharmacological therapy, can promote a reduction in individual cardiovascular risk and in major cardiovascular events.

However, the cardioprotective effect of PUFA has been questioned in a few meta-analyses [121,122]: different dietary habits of the studied populations have been highlighted (the intake of omega-3 PUFA is 15 times less in Europe compared to Eastern Countries, such as Japan), as well as different doses and follow up periods, and these biases might have determined mixed results [123].

In the past few years, two intervention studies have analysed the effect of omega-3 PUFA. In the REDU-CE-IT study, involving approximately 8000 patients, the authors evaluated the supplementation with eicosapent ethile, a high resistance purified formulation of EPA, on triglycerides reduction and on cardiovascular events in association with statin therapy. Study participants were patients with cardiovascular disease, diabetes or other cardiovascular risk factors, with triglycerides levels ranging from 150 to 500 mg/dL and LDL cholesterol ranging from 40 to 100 mg/dL. The intervention group received 4 g EPA every day and, after a 4.8 year follow up, showed a 25% reduction in the primary endpoints, that is to say, a compound of cardiovascular death, non-fatal myocardial stroke or cerebral stroke, coronary revascularisation or unstable angina [124]. The STRENGHT Study (Cardiovascular Outcomes with Omega-3 Carboxylic Acids (Epanova) in Patients with High Vascular Risk and Atherogenic Dyslipidaemia) was designed to evaluate the effect of DHA plus EPA in patients at high cardiovascular risk, in association with statin therapy, but it was prematurely discontinued for the lack of end points achievement [125].

There are few data on PUFA effect on lipid profile in the paediatric population. ESPGHAN has recently evaluated the positive effect of PUFA on global health in children [126], whereas PUFA effect on lipoproteins in children with dyslipidaemia were described by Engler et al. in the EARLY study [127]. Dangardt et al. reported an improvement in vascular function and a reduction in inflammation in obese adolescents treated with omega-3 PUFA [128], whereas Nobili et al. documented a reduction in hepatic steatosis in children with non-alcoholic fatty liver disease [129].

Gidding S et al. reported a reduction in triglycerides levels in children and adolescents (42 subjects, mean age 14 years) treated with fish oil containing 4 g/day DHA and EPA, with no significant LDL-cholesterol modification but with a significant anti-thrombotic effect (reduction in plasmatic fibrinogen and in plasminogen activator) [130].

Del Bò et al. recently documented an increase in omega-3 and omega-6 PUFA in erythrocyte cell membranes in paediatric patients with dyslipidaemia on dietary treatment with good compliance; patients were given hemp (3 g/day 1.4 g linoleic acid, 0.7 g di alpha linolenic acid) for eight weeks, with a 14% reduction in LDL-cholesterol levels compared to the control group [131]. Further studies are needed to consolidate this evidence.

PUFA have few adverse effects and a good safety profile, but they are derived from fish, and their taste is not always well accepted. Seaweed derived omega-3 PUFA should be considered in order to obtain better compliance.

### 6.2. Curcumin

Curcumin is the main phenolic compound of the aromatic rhizome of an Asian plant, *Curcuma domestica* (or *C. longa*), belonging to the ginger family. Curcumin has multiple properties, including cholesterol-lowering activity, anti-oxidative and anti-inflammatory effects [132]. The lipid-lowering mechanism of curcumin are unclear: curcumin seems to inhibit NPC1L1 transporter expression through transcription factor SREBP2 [133] and to enhance cholesterol efflux through ABCA1 activation [134]. Curcumin can also increase the number of LDL receptors through inhibition of PCSK9 activity [135] and modulate microRNA [136]. The effects of curcumin on lipid profile are not always univocal: in a meta-analysis conducted by Sahebkar et al., curcumin was not associated with significant modifications of LDL cholesterol, HDL cholesterol or triglycerides [137], whereas in another study involving patients with metabolic syndrome who were given curcumin 1 g/day, LDL cholesterol, triglycerides and Lp(a) were all reduced, and HDL cholesterol increased [138]. These results have been confirmed in other trials that have also highlighted an anti-diabetic effect of turmeric [139].

Curcumin has a good safety profile, but it has low bioavailability, as it is slightly water-soluble; thus, new formulations are nowadays under evaluation to facilitate its use [140].

No data are yet available in paediatric patients.

## 7. Nutraceuticals in Combined Therapy

Scientifical evidence that supports the cholesterol-lowering effect of some nutraceuticals has led to the development of multi-activity products (foods or supplements) containing different bioactive compounds so as to obtain an additive lipid-lowering action. The use of nutraceuticals with multiple metabolic actions on cholesterol metabolism brings about an improvement in cardiovascular risk profile, in particular in primary prevention for those patients with low-to-moderate hypercholesterolaemia not on target, as well as in patients with statin-associated side effects, who cannot be treated with high statin doses [141].

The association of natural products with different mechanisms of action can potentiate their effect, acting simultaneously on different biochemical pathways: they can reduce intestinal cholesterol absorption and/or increase cholesterol excretion (soluble fibres, glucomannan, phytosterols, probiotic), or they can increase cholesterol hepatic re-uptake (soy, berberine) or they can inhibit HMGCoA reductase activity, thus limiting endogenous cholesterol synthesis (monacolins, polycosanols, soy and bergamot) [71].

There are many associations of nutraceuticals: red yeast rice and polycosanols, red yeast rice, polycosanols and berberine, red yeast rice and phytosterols, and so on. These associations have been tested with good results in adult patients, but there are few data for paediatric patients. The available evidence in paediatric patients seems promising, but further and more robust studies are needed on this topic.

## 8. Nutraceuticals and Pharmacologic Therapy

Many studies have highlighted an additive and complementary effect of nutraceuticals both on lifestyle and pharmacological therapy. Their association with the lipid-lowering drugs could help achieve the target lipid plasma levels with lower dosages and fewer adverse effects [142]. Nutraceuticals can be used in combined therapy, especially in those patients who do not tolerate a high dose of statins, with poor adherence to the therapy [143].

The main associations proposed are statin and PUFA; statin and fibres; ezetimibe, red yeast rice, polycosanols and berberine; statin and silymarin; ezetimibe and silymarin; statin/ezetimibe and phytosterols. However, it is important to acknowledge that these associations have been tested in the short-term and limited cohort studies, and their long-term efficacy is not yet fully convincing. Moreover, further studies are needed before adopting these associations in paediatric patients.

## 9. Final Considerations

The use of nutraceuticals with cholesterol-lowering effect, both as functional foods and as supplements, is an interesting strategy for paediatric patients, but it may have some risks. As a matter of fact, trials on nutraceuticals have been frequently carried out on a limited study population, so further multicentric studies on larger cohorts are needed (Table 3).

In clinical practice, the availability of nutraceuticals as supplements without medical prescription could result in uncontrolled use such as auto-prescription, therapy discontinuation and/or excessive dose, with a consequent reduced therapeutic effect and/or increased adverse events. However, we must not forget that the effects on lipid profile are closely related to their continuous intake within a defined therapeutic programme [144].

Furthermore, functional foods are expensive compared to traditional foods and drugs, and this could be a major limitation for their long-term use [145].

In conclusion, paediatric lipidologists should consolidate a good alliance with their patients so as to avoid improper and uncontrolled use of nutraceuticals.

Despite the fact that randomised controlled trials on nutraceuticals use in paediatric patients are few and conducted on a limited number of patients, a beneficial short- and medium-term effect on lipid profile has been observed, especially with regard to total- and LDL-cholesterol plasma level reduction. Soluble fibres and phytosterols are the most studied nutraceuticals in children, as they seem to be well tolerated with no relevant adverse effects.

According to the available scientific evidence and to EAS guidelines, we can suggest the use of functional foods containing fibres and phytosterols for children with genetic dyslipidaemia, starting from six years of age [6].

Nutraceuticals containing fibres and phytosterols should always be considered as a complement to the dietary [146] and lifestyle intervention, which remains the milestone approach in the treatment of dyslipidaemia in paediatric patients [147]. Nutraceuticals should be used for a short period and in those children who do not tolerate pharmacological therapy or who cannot yet receive it due to age limitations.

## Figures and Tables

**Table 1:** Nutritional and lifestyle intervention in paediatric patients with hypercholesterolaemia; adapted from Giovannini et al. [5] and Catapano et al. [6].

Nutritional treatment is the milestone intervention in paediatric patients at increased CVD risk. Dietary-nutritional intervention in children with dyslipidaemia has the main objective to establish correct eating habits that are most likely to be maintained over time until adulthood. It is recommended to limit the consumption of foods with high content of saturated fats, as they are the main responsible for the increase in cholesterolaemia. A prudent low-fat diet is recommended, encouraging the intake of fruits, vegetables, unrefined grains, pulses, fish and meats. The traditional Mediterranean diet represents the ideal model because it proposes a diet rich in these foods, reduced consumption of salt and condiments in the preparation of foods, a preference for extra virgin olive oil and steamed, baked and stewed foods. Physical activity should be promoted, and conditions related to the CVD risk should be limited, such as sedentary life, cigarette smoke (also passive), obesity, hypertension and diabetes.

**Table 2:** Nutraceuticals effect on lipid profile.

	Reduce Total Cholesterol	Reduce LDL Cholesterol	Reduce Triglycerides	Increase HDL Cholesterol
**Fibres**	+	+		
**Phytosterols and stanols**	+	+		
**Probiotics**	+	+		
**Red yeast rice**	+	+		
**Soy and lupins**	+	+	+	+
**Omega-3 fatty acids**			+	

+ means positive effect.

**Table 3:** Characteristics of studies about the effect of nutraceuticals in paediatric subjects with dyslipidaemia.

Nutraceuticals	Type of Study	Aim	Casuistry	Dose and Duration of the Intervention	Effects	Reference
**Psyllium**	DB-CO- RCT	Lipid-lowering effect of cereals added to psyllium	32 children Age: 6–18 years Inclusion criteria: LDL-C ≥ 90th percentile	8-week diet: 58 g of cereals added to psyllium (6.4 g) or to placebo	↓ TC: −5% ↓ LDL-C: −6.8%	Davidson MH et al., Am J Clin Nutr 1996 [36]
	RCT	Reduction of CT and LDL-C after integration with psyllium	36 children Age: 3–17 years Inclusion criteria: FH	Age ≤ 7 years: 5 g/die Age ≥ 7 years: 10 g/die Duration: 8.0 ± 1.1 months	↓ TC: −18% ↓ LDL-C: −23%	Glassman M et al., AJDC 1990 [37]
	SB-RCT	Effectiveness of psyllium in CT and LDL-C reduction	50 children Age: 2–11 years Inclusion criteria: LDL-C ≥ 110 mg/dL	CHILD I: all groups. Intervention: cereals containing 3.2 g of psyllium. Duration: 12 weeks	↓ TC: −9.6% ↓ LDL-C: −15.7% ↑ HDL-C: +9.96%	Williams CL et al., J Am Coll Nutr 1995 [38]
	DB-RCT	Effectiveness of psyllium on LDL-C in Brazilians children and teenagers with dyslipidaemia	51 children Age: 6–19 years Inclusion criteria: TC ≥ 175 mg/dL	CHILD II: 6 weeks. Intervention group: 7 g/die of psyllium Control group: 7.0 g/die of cellulose	↓ TC: −7.7% ↓ LDL-C: −10.7%	Ribas SA et al., Br J Nutr 2015 [39]
**Glucomannan**	DB-CO- RCT	Efficacy and tolerability of supplementation with glucomannan	36 FH children Age: 6–15 years Inclusion criteria: baseline values of CT > 90°percentile by gender and age	CHILD I diet Duration: 4 weeks 2 cps/day of Glucomannan or placebo Duration: 8 weeks Wash-out: 4 weeks	↓ TC: 5.1% ↓ LDL-C: 7.3%	Guardamagna O et al., Nutrition 2013 [40]
	DB-RCT	Lipid-lowering effects of glucomannan in combination with CP or PC	132 children Age: 3–16 years Inclusion criteria: TC ≥ 170 mg/dL, 1 parent with CT 240 mg/dL, or familiarity for CVD	Randomised assignment to 5 neutraceuticals and 1 placebo (only resistant starch) 8-week treatment groups Duration: 8 weeks	GM + CP: ↓ LDL-C: −16% GM + PC: ↓ LDL-C: −10%	Martino F et al., Atherosclerosis 2013 [41]
**Phytosterols and stanols**	DB-CO-RCT	Lipid-lowering effects of sterols	30 children Age: 6–9 years Inclusion criteria: TC ≥ 170 mg/dL, LDL-C ≥ 110 mg/dL	Intervention group: milk +1.2 g/day of sterols vegetable. Control group: skim milk Duration: 8 weeks	Intervention group: ↓ TC: −4.5% ↓ LDL-C: −11.1% Control group: TC: +1.4 ↓ LDL-C: −0.9%	Ribas SA et al., NMCD 2017 [50]
	DB-CO-RCT	Lipid-lowering effects of stanols	38 children Age: 7–12 years Inclusion criteria: “Definite” diagnosis or “possible” diagnosis of FH	CHILD I + 1.6 g of stanols or placebo Duration: 8 weeks	↓ LDL-C 10.2% ↓ TC and ApoB 7.4%	Amundsen AL et al., Am J Clin Nutr 2002 [51]
	CT	Effects of sterols on LDL-C levels	64 children 25 LDL-C ≥ 130 mg/dL 34 LDL-C ≤ 130 mg/dL Age: 4.5–15.9 years	CHILD II Intervention group: yoghurt (2 g/die sterols) Duration: 6–12 months	Intervention group: ↓ LDL-C: −13%	Garoufi A et al., IJP 2014 [52]
**Red yeast rice**	DB-CO-RCT	Efficacy and safety of a combination of red yeast rice extract and policosanols	80 children Age: 8–16 years	CHILD I red yeast rice 200 mg/die + policosanols 10 mg/ placebo Duration: 8 weeks Wash-out: 4 weeks	↓ TC: 18.5% ↓ LDL-C: 25.1% ↓ ApoB: 25.3%	Guardamagna O et al., NMCD 2011 [71]
**Soy**	RCT	The effect of integration with protein of soy on lipoproteins	23 children Age: 4–18 years Inclusion criteria: FH	Step 1: diet Duration: 3 months Step 2: diet + soya protein 0.25 g/kg in replacement animal protein. Duration: 3 months	Step 1: ↓ TC: −12.3% ↓ LDL-C: −11.8% ↓ ApoB: −10.6% Step 2: ↓ TC: −7.7% ↓ LDL-C: −6.4% ↓ ApoB: −12.6%	Weghuber D et al., Br J Nutr 2008 [104]
	RCT	The effect of soy on LDL-C levels	17 children Age: 5–13 years Inclusion criteria: FH	Soy group: soy-enriched fat modified diet Control group: fat modified diet Duration: 13 weeks	LDL-C decrease: statistically significantly greater in the soy group	Helk O et al., Clin Nutr 2020 [105]
**Omega-3 polyunsaturated long-chain fatty acids**	DB-CO-RCT	The efficacy of fish oil in lowering TG and impacting lipoprotein particles	42 children Age: 10–17 years Inclusion criteria: TG ≥ 150 mg/dL and <750 mg/dL, LDL-C <160 mg/dL	Intervention group: 4 g daily of fish oil Control group: placebo Duration: 8 weeks	TG decrease: greater in the intervention group	Gidding SS et al., J Pediatr 2014 [130]
	RCT	The effectiveness of hempseed oil in the modulation of hyperlipidaemia and evaluation of fatty acid composition of red blood cells	36 children Age: 6–16 years Inclusion criteria: hyperlipidaemia primitive and compliance to the alimentary indications	Control group: CHILD I Intervention group: hempseed oil 3 g/die Duration: 8 weeks	Intervention group: ↓ RC SFA: −5.02% ↓ RC MUFA: −2.12% ↑ PUFA *n* − 3: + 1.57% ↑ PUFAs *n* − 6: +5.39% ↑ Omega 3 Index: 1.18% ↓ LDL-C: 14.2%, Control group: ↓ LDL-C: −4.94%	del Bo’ C et al., Food Res Int. 2019 [131]

DB—double blind; SB—single blind; CO—cross-over; RCT—randomised controlled trial; CT—controlled trial; ↓—decrease; ↑—increase; FH—familial hypercholesterolaemia; TC—total cholesterol; LDL-C—low-density lipoprotein cholesterol; HDL-C—high-density lipoprotein cholesterol; TG—triglycerides; GM—glucomannan; CP—chromium-polynicotinate; RC—blood red cells; SFA—saturated fatty acids; MUFA—monounsaturated fatty acids; PUFA—polyunsaturated fatty acids.

## Data Availability

Not applicable.

## Abbreviations

AAP	American Academy of Pediatrics
AHA	American Heart Association
CHD	Coronary heart disease
DHA	Docosahexaenoic acid
EAS	European Atherosclerosis Society
EFSA	European Food Safety Authority
EPA	Eicosapentaenoic acid
EU	European Union
FCHL	Familial combined hypercholesterolaemia
FH	Familial hypercholesterolaemia
HDL	High-density lipoprotein
HMGCoA	HydroxyMethylGlutaryl CoA
LDL	Low-density lipoprotein
NCEP	National Cholesterol Education Programme
NPC1L1	Niemann–Pick C1-like protein
PUFA	Polyunsaturated fatty acids
RYR	Red yeast rice
SCFA	Short-chain fatty acids
TG	Triglycerides
